# P-55. Knowledge of Respiratory Syncytial Virus (RSV) Vaccine in Pregnant Women: A survey among Healthcare Professionals

**DOI:** 10.1093/ofid/ofae631.262

**Published:** 2025-01-29

**Authors:** Leticia Santoyo, Karina López, Michel F Martínez-Reséndez, Gloria Mayela Aguirre-García, Erick Garza

**Affiliations:** TECSALUD HEALTH SYSTEM, Monterrey, Nuevo Leon, Mexico; TECSALUD HEALTH SYSTEM, Monterrey, Nuevo Leon, Mexico; Instituto Tecnológico y de Estudios Superiores de Monterrey, School of Medicine and Health Sciences, Monterrey, Nuevo Leon, Mexico, Monterrey, Nuevo Leon, Mexico; TecSalud, Monterrey, Nuevo Leon, Mexico; TECSALUD HEALTH SYSTEM, Monterrey, Nuevo Leon, Mexico

## Abstract

**Background:**

Respiratory Syncytial Virus (RSV) is a leading cause of acute lower respiratory tract infection (RTI), playing a significant role in infant mortality under six months of age, especially in low and middle-income countries. In August 2023, the U. S. Food & Drug Administration approved active immunization of pregnant women between 32 to 36 weeks gestational age for the prevention of lower RTI and severe lower RTI caused by RSV in infants up to 6 months of age.

Figure 1.

**Figure:**
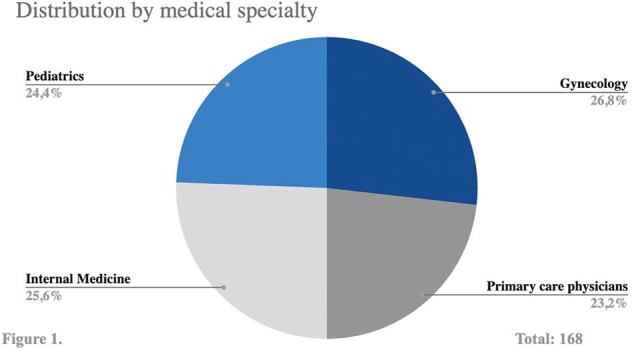

Distribution of responses according to medical specialt

**Methods:**

An electronic survey comprised of 18 questions was administered to primary care physicians, internists, gynecologists, and pediatricians. The survey included 4 items to gather participant characteristics, 1 addressing general vaccination recommendations during pregnancy, 5 covering general aspects of the RSV vaccine, 7 focusing on knowledge of the RSV vaccine during pregnancy and 1 item to ascertain whether they recommend this vaccine. If participants lacked prior knowledge of the vaccine or its use in pregnant patients, the survey was concluded

Figure 2.

**Figure:**
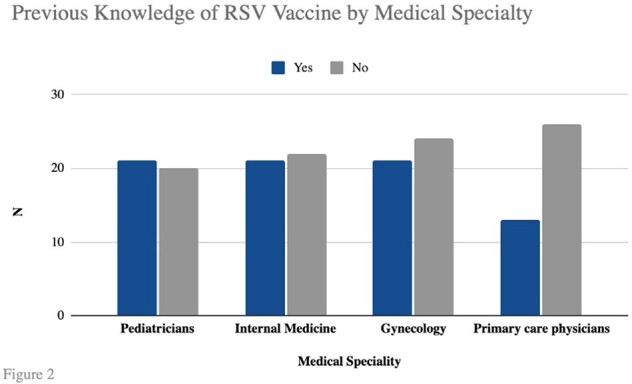

Previous Knowledge of RSV Vaccine by Medical Speciality

**Results:**

168 responses were received, the distribution by specialties is shown in **Figure 1**. A total of 76/168 doctors (45.2%) were aware of the existence of a vaccine against RSV **(Figure 2),** 52/76 (68.4%) had knowledge of the vaccine targeted to pregnant women **(Figure 3)**. Among the doctors familiar with this vaccine, 29/52 (55.8%) knew that it is a single-dose vaccine. Additionally, 28/52 (53.8%) were aware of when it should be administered, 31/52 (59.6%) knew it is a seasonal vaccine and 27/52 (51.9%) were previously aware of its efficacy in providing protection to infants up to 6 months old. Furthermore, 100% of the surveyed expressed that they would recommend the vaccine once it is approved in Mexico

Figure 3.

**Figure:**
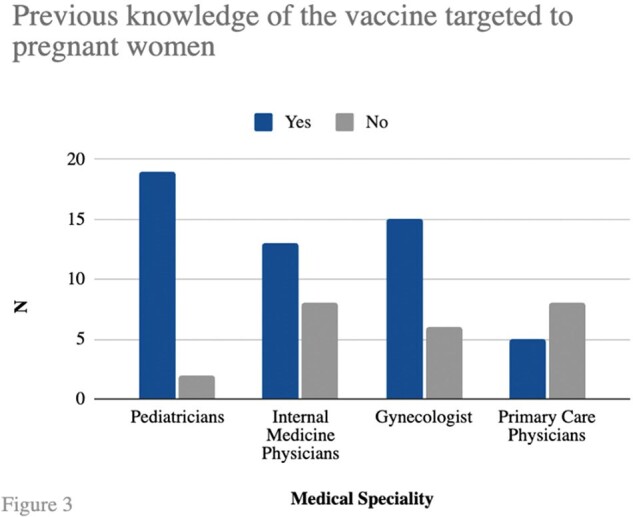

Previous knowledge of the vaccine targeted to pregnant women

**Conclusion:**

Only 27/168 (16%) of the surveyed demonstrated a satisfactory knowledge about the RSV vaccination in pregnant women, including details such as dosage, indication, timing of administration and efficacy.

These surveys serve to assess physicians knowledge on the topic under study, and are valuable educational tools for the respondents by provifing information and reliable sources for further reading of the topic. Nevertheless our findings highlight the need for additional comprehensive educational campaigns targeting healthcare providers

**Disclosures:**

**All Authors**: No reported disclosures

